# Does orthognathic surgery affect mandibular condyle position? A retrospective study

**DOI:** 10.1007/s10006-023-01181-3

**Published:** 2023-09-23

**Authors:** Bronislava Dvoranova, Michal Vavro, Ladislav Czako, Dusan Hirjak

**Affiliations:** 1 Department of Oral and Maxillofacial Surgery, Faculty of Medicine, Comenius University and University Hospital Bratislava, Bratislava, Slovakia; 2 Department of Stomatology and Maxillofacial Surgery, Faculty of Medicine, Comenius University, Oncologic Institute of St Elisabeth, Bratislava, Slovakia

**Keywords:** Orthognathic surgery, Temporomandibular joint, Condylar sag, Bicortical screws, Miniplates

## Abstract

**Purpose:**

The aim of this study is to analyze mandibular condyle position changes after bilateral sagittal split osteotomy (BSSO) and bimaxillary orthognathic surgery in patients operated at a single department by two surgeons in 2013–2022. Compared were groups of mandibular advancement vs setback and bimaxillary vs BSSO.

**Methods:**

Ninety-nine subjects were included. Inclusion criteria were patients who underwent one of the BSSO or bimaxillary surgery and had CT scans performed before and after surgery. Preoperative CT scans were performed 1 day before surgery and postoperative CT scans 6–12 months afterwards. Changes in mandibular condyle position were measured in axial and sagittal planes.

**Results:**

CT condylar position measurements indicated significant postoperative changes in AB angle bilaterally (*p* =  < 0.001). In mandibular advancement and setback comparison, values were significantly lower in ABL angle values in the setback group (*p* = 0.011326) and significantly higher in FDR in the advancement group (*p* = 0.005795). There were no statistically significant changes found in BSSO and bimaxillary group comparison.

**Conclusion:**

Within the limitations of this study, it can be concluded that orthognathic surgery does have a moderate effect on position of the condyles, especially condylar rotation in transversal axis.

## Introduction

Orthognathic surgery covers a wide range of techniques used to correct maxillofacial occlusal discrepancies and facial deformities. Most orthognathic surgical procedures consist of a mandibular and/or maxillary osteotomy followed by segment fixation in a new, preplanned position. In preoperative planning, facial harmony, airway, occlusion, and temporomandibular joint (TMJ) function are taken into consideration.

One of the most frequently used techniques is bilateral sagittal split osteotomy (BSSO) developed by Obwegeser and Trauner [1]. BSSO is a surgical method in which the mandibular ramus is segmented and placed in the ideal position in relation to the maxillomandibular complex. Internal fixation (IF) is used to stabilize the segments. Different systems of IF have been described—bicortical or monocortical screws and miniplates are most frequently used. Changes to the postoperative condylar position should be avoided to limit adverse postoperative effects (i.e., condylar resorption, functional disorder, and relapse) and to ensure correct mandibular function. Proper condylar seating is essential for appropriate postoperative results, long-term skeletal stability, and TMJ function. Central or peripheral condylar sag may cause malocclusion immediately after the removal of perioperative maxillomandibular fixation (MMF) or later in the postoperative period. It can also lead to skeletal relapse and condylar resorption [2].

It is important that the technique of mandibular osteosynthesis does not displace or rotate the condyle. Proper surgical technique should result in only minimal condylar remodeling. Selecting between rigid internal fixation (RIF)—bicortical screws and semirigid fixation—monocortical screws and miniplates still remains controversial. Leaving necessary gaps between the segments and removing any bony interferences to avoid segment displacement is essential to facilitate postoperative skeletal stability [3].

All modes of mandibular osteosynthesis utilized in orthognathic surgery can influence the condyle position and postoperative TMJ function. Improper postoperative condylar position can result in TMJ dysfunction, e.g., disk displacement and condylar resorption in the future [4–7].

The aim of this retrospective study was to evaluate condylar position changes measured on CT scans after various types of orthognathic surgery (BSSO only, bimaxillary surgery) and to compare the impact of two types of mandibular movement on the TMJ—mandibular advancement and setback.

## Patients and methods

Ninety-nine subjects were analyzed. Inclusion criteria were that the orthognathic surgery was performed at a single department by two surgeons (DH, MV) and the CT scans for each case were made before and minimum 6 months after surgery.

### Condylar position

CT scans (Somatom Plus 4, Siemens, Germany) were performed utilizing 1-mm axial and sagittal slices. Preoperative scans were taken one day before surgery. Postoperative scans were taken between 6 and 12 months after surgery.

For condyle position evaluation, a method introduced by Harris and later modified by Lee and Park was utilized [8, 9]. In axial scans, a slice with the greatest mediolateral dimension of the condyles was identified. Midsagittal line was defined, connecting the base of vomer to the midpoint of clivus of the sphenoid bone (A line). An axis was then determined for each of the condyles, and the angle between both axes and the A line was measured (AB angle, measured in degrees).

**Fig. 1:**
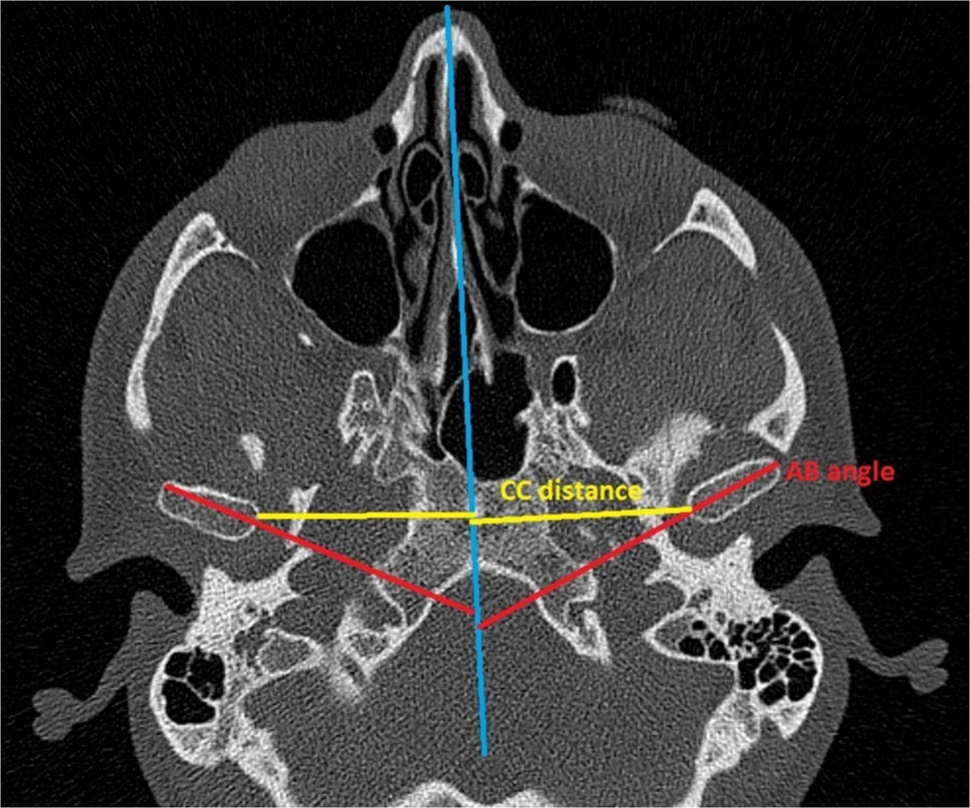
Axial CT condylar position parameters

C lines were specified—lines perpendicular to A line connecting the most medial point of each condyle to the A line. This distance was measured in millimeters and labeled CC distance (Fig. 1).

In sagittal scans, a slice was found where both the articular eminence and external acoustic meatus were best visualized. Line D was set, connecting the most cranial point of external acoustic meatus (porion) to the most caudal point of articular eminence. E line was set parallel to D line, utilized running through the superior surface of mandibular condyle. The ED distance was then measured in millimeters.

**Fig. 2:**
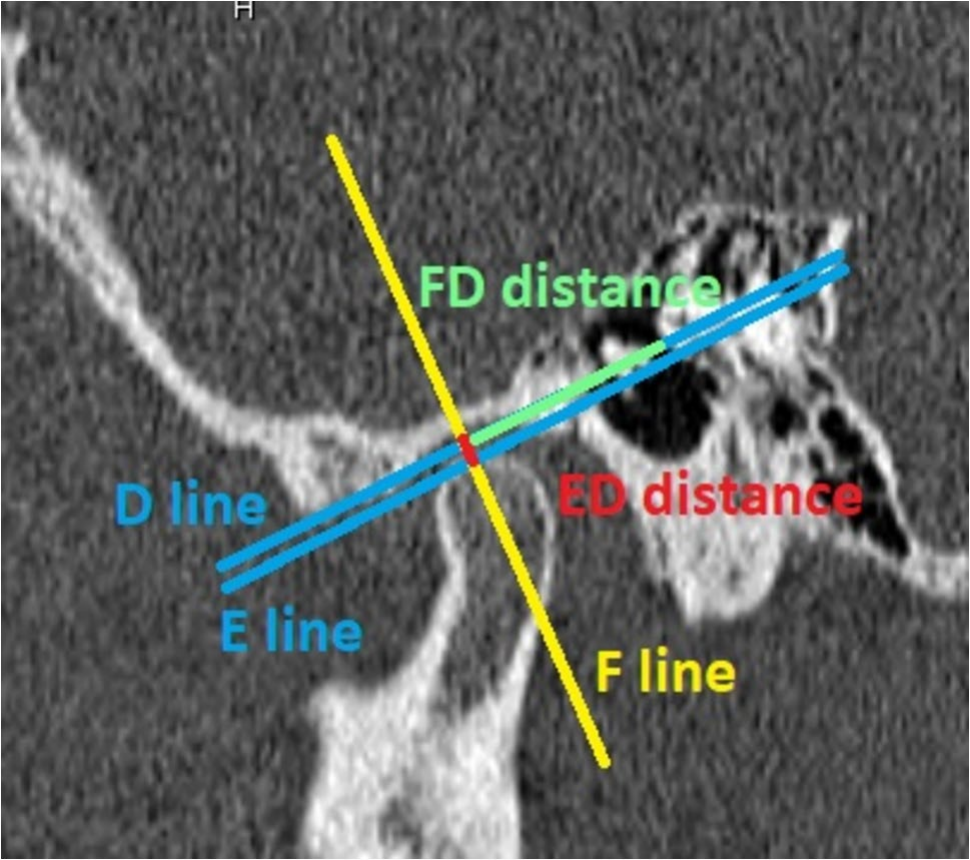
Sagittal CT condylar position parameters

Line F was determined perpendicular to line D, running through the most cranial point of mandibular condyle. The distance between F line and porion (FD distance) was measured in millimeters (Fig. 2).

All the measurements on sagittal scans were performed bilaterally. Pre- and postoperative results were then compared to quantify the positional changes.

## Results

There were 79 women (79.8%) and 20 men (20.2%). Average patient age was 27.5 (± 6.5 years). Average length of hospital stay was 5.5 (± 1.4 days). A III malocclusion was present in 57 patients (57.6%), A II malocclusion in 32 patients (32.3%), and laterogenia in 12 patients (12.1%). Vertical malocclusion—skeletal open bite was present in 12 patients (12.1%). The complete number exceeds 100%, since 14 patients presented with a combination of the previous diagnoses (14.1%).

BSSO only was performed in 51 patients (51.5%). Bimaxillary surgery was performed in 48 patients (48.5%). Average operative time of BSSO was 112.73 ± 26.51 min. Average operative time of bimaxillary surgery was 203.56 ± 42.38 min.

Forty-three patients underwent mandibular advancement (43.4%) and 56 patients underwent mandibular setback (56.6%). Symmetrical movement of the distal mandibular segment was performed in 43 patients (43.4%) and asymmetrical movement in 56 patients (56.6%). The average mandibular distal segment movement range for BSSO was 4.69 ± 2.34 mm on the right side and 5.27 ± 17 mm on the left side. The average mandibular distal segment movement range for bimaxillary surgery was 4.21 ± 2.3 on the right side and 4.33 ± 2.05 on the left side.

A paired *t*-test was used for analysis of condylar position changes. There was a statistically significant difference found in the AB angle bilaterally (right, *p* =  < 0.001; left, *p* =  < 0.001) (Table 1).

**Table 1:** Condylar position pre- and postoperative values’ comparison *t*-test

Parameter	Average change	Confidence interval lower limit	Confidence interval upper limit	*p* value
CCL	− 0.29697	− 0.74309	0.149147	0.19
CCR	0.379798	− 0.00685	0.766444	0.0541
ABL	− 2.65859	− 4.08628	− 1.23089	3.6e − 04
ABR	− 4.62727	− 6.0639	− 3.19064	5.6e − 09
EDL	− 0.07061	− 0.30955	0.168334	0.559
EDR	0.090404	− 0.25736	0.438167	0.607
FDL	0.031919	− 0.41849	0.48233	0.888
FDR	0.262828	− 0.1169	0.642559	0.173

Average value changes of mandibular advancement and mandibular setback patients were compared using the ANOVA test (in EDL parameter, the Kruskal–Wallis test was used because of variance inequality). There was a significant difference found in ABL value changes (*p* = 0.011326)—in the setback group, the values were significantly lower. There was also a difference in FDR value changes—in the advancement group, the values were significantly higher (*p* = 0.005795) (Table 2)

**Table 2:** Comparison of advancement and setback groups in condylar position changes (ANOVA, Kruskal–Wallis in EDL)

Parameter	Advancement group average change (43 patients)	Setback group average change (56 patients)	*p* value
CCL	− 0.01667	− 0.56078	0.298289
CCR	0.48125	0.284314	0.367259
ABL	− 2.275	− 3.01961	0.011326
ABR	− 5.06042	− 4.21961	0.169629
EDL	− 0.20438	0.055294	0.5579
EDR	− 0.13688	0.304314	0.295643
FDL	0.023958	0.039412	0.963936
FDR	0.309583	0.218824	0.005795

Average value changes of bimaxillary patients and BSSO and maxilla only patients were compared using the ANOVA test (in the CCL parameter, the Kruskal–Wallis test was used because of variance inequality). No statistically significant changes were found (Table 3)

**Table 3:** Comparison of bimaxillary and BSSO groups in condylar position changes (ANOVA, Kruskal–Wallis in CCL)

Parameter	Bimaxillary group average change (48 patients)	BSSO group average change (51 patients)	*p* value
CCL	− 0.01667	− 0.56078	0.3033
CCR	0.48125	0.284314	0.615944
ABL	− 2.275	− 3.01961	0.607505
ABR	− 5.06042	− 4.21961	0.564282
EDL	− 0.20438	0.055294	0.283388
EDR	− 0.13688	0.304314	0.209969
FDL	0.023958	0.039412	0.973063
FDR	0.309583	0.218824	0.814007

## Discussion

Changes of condylar position after orthognathic surgery may in long term lead to skeletal relapse, malocclusion, condylar resorption, and TMJ dysfunction. Reyneke [2, 10] described various types of condylar displacement—condylar sag—and divided it into two main categories—peripheral and central. Reyneke also suggested that assuring the proper relationship of the condyle to the glenoid fossa is critical and is probably the most demanding step in the BSSO procedure.

Condyle positioning appliances were introduced to address this issue. However, in the authors’ department, these appliances have not yet been utilized. Also, usage of these devices remains controversial, as the benefit of using them is uncertain [11, 12]. Some studies also suggest computer-assisted condylar positioning techniques. In a systemic review, Chow reports considerable accuracy in both the non-computer-assisted and computer-assisted condylar positioning techniques [13].

The amount of segment movement, local anatomy, presence of eventual bony interferences, and experience of the performing surgeon also influence condylar position. Harris reported the amount of distal segment movement, extent of mandibular rotation, and local anatomy indication the shape of the mandible may be important [8].

Type of osteosynthesis can also be an important factor influencing condylar position after orthognathic surgery. Some authors still recommend bicortical screws, even despite the reported condylar displacement risk and higher percentages of skeletal relapse [14]. Some authors prefer the usage of one miniplate with monocortical screws, while others apply two miniplates [3, 15, 16]. Reyneke believes the use of bicortical screws for BSSO osteosynthesis relates to a higher risk of peripheral condylar sag—when the segments are forcefully fixated, tension is applied to the condyle, which can result in condylar displacement medially, laterally, or inferiorly [10]. In the authors’ earlier published studies, no significant changes in condylar position and function were found after fixing BSSO with bicortical screws [17, 18].

Verhelst stated it is believed that BSSO induces biomechanical stresses at the TMJ [19]. This biomechanical stress can lead to a process called “physiological joint remodeling.” When joint remodeling surpasses its physiological limits, pathological remodeling can occur regardless of whether the patient had preexisting TMJ dysfunction. The question of why one overloaded joint develops TMD and another develops condylar resorption remains an enigma. In the same article, the authors stated that in the postoperative phase, three types of biomechanical stresses are possible at the TMJ—prolonged alteration of the condyle position, tension in the muscles attached to the mandible, and postoperative orthodontic forces incurred with the use of elastics.

Mild condylar remodeling is normal after orthognathic surgery. In a study published by Claus utilizing CBCT condylar superimposition, medial and lateral surfaces presented fewer bone changes. Overall, the bone changes in their study were smaller than 1 mm in 85.7% [20]. In a volumetric CBCT study of the TMJ published by Park, condylar volume and height showed a significant decrease in 6 months after bimaxillary orthognathic surgery, but a significant increase between 6 months to 6 years after surgery [21]. Another study published by Xi [22] also found a mean of 6.1% in postoperative condyle volume. The question of follow-up timing plays an important role in the whole study model. As mentioned in previous studies [19, 21, 22], follow-ups are recommended immediately after surgery, at 6 months after surgery, and also after longer periods of time [21] in order to properly characterize the extent of condylar remodeling and changes of position after orthognathic surgery.

All of the cases in this study were performed using conventional planning. Digital planning has been utilized in the authors’ department since April 2023, and none of the patients operated have yet managed it to the follow-up CT after 6–12 months after surgery. Three-dimensional planning offers more possibilities and methods in assessing TMJ position pre- and postoperatively and also more detailed planning of the osteotomies, movements, and also segment torque and flaring in order to avoid abnormal movements [23, 24].

In this study, the results showed significant changes in pre- and postoperative values in AB angle, which represents condylar rotation in the transversal axis. This change was even more apparent in mandibular setback group, where the values showed a significant decrease, indicating medial condylar rotation. Higher FDR values in the advancement group indicate a more anterior postoperative condylar position in comparison to the setback group.

There is still controversy as to whether orthognathic surgery influences TMJ function. Farella reports an unpredictable and variable occurrence of TMD after orthognathic surgery [4]. In Abrahamsson’s study, TMD symptoms after orthognathic surgery improved [5]. Moroi reports an improved occlusal contact area, bite force, and occlusal balance after orthognathic surgery [25]. In a study published by Togashi, it was reported that 16.5% of the patients with no preoperative TMJ disorder symptoms started to experience TMJ disorders after orthognathic surgery [6]. Kretschmer reported a 6.4% incidence of TMJ pain, 4.8% crepitus, and clicking 19.1% developing after orthognathic surgery in preoperatively asymptomatic patients [7].

In a systematic review published by Veldhuis, also confirmed by other authors [5, 17–19, 25], it was concluded that orthognathic surgery does not seem to have any harmful impact on the TMJ [17–19, 25, 26].

The authors of this study can conclude that orthognathic surgery does have a moderate effect on postoperative position of the condyles, especially regarding condylar rotation in the transversal axis.

## Data Availability

Data set is available at Synapse: https://doi.org/10.7303/syn52386226
