# Correction to: Does orthognathic surgery affect mandibular condyle position? A retrospective study

**DOI:** 10.1007/s10006-024-01257-8

**Published:** 2024-05-03

**Authors:** Bronislava Dvoranova, Michal Vavro, Ladislav Czako, Dusan Hirjak

**Affiliations:** 1 Department of Oral and Maxillofacial Surgery, Faculty of Medicine, Comenius University and University Hospital Bratislava, Bratislava, Slovakia; 2 Department of Stomatology and Maxillofacial Surgery, Faculty of Medicine, Comenius University, Oncologic Institute of St Elisabeth, Bratislava, Slovakia

**Correction to: Oral and Maxillofacial Surgery (2023)**

10.1007/s10006-023-01181-3

The original article contains a numerical error in Table 2. Corrected Table 2 provided:

**Table:** 

Parameter	Advancement Group Average Change (43 patients)	Setback Group Average Change (56 patients)	*P* value
CCL	−0,565	−0,091	0,298289
CCR	0,581	0,225	0,367259
ABL	−0,598	−4,241	0,011326
ABR	−5,765	−3,754	0,169629
EDL	−0,026	−0,105	0,74881
EDR	−0,120	0,252	0,295643
FDL	0,044	0,023	0,963936
FDR	−0,333	0,720	0,005795

Also, because of the numerical error, there has been a misinterpretation of the results. Hence, in the Abstract and Results section, the results are as following:

**In Abstract:**

**Results:** CT condylar position measurements indicated significant postoperative changes in AB angle bilaterally (*p* = <0,001). In mandibular advancement and setback comparison, values were significantly lower in ABL angle values in setback group (*p* = 0,011326), and significantly lower in FDR in advancement group (*p*=0,005795). There were no statistically significant changes found in BSSO and bimaxillary group comparison.

**In Results:**

There was also a difference in FDR value changes – in advancement group, the values were significantly lower (*p* = 0,005795) (Table 2).

**In Discussion:**

In this study, the results showed significant changes in pre- and postoperative values in AB angle, which represents condylar rotation in the transversal axis. This change was even more apparent in mandibular setback group, where the values showed a significant decrease, indicating medial condylar rotation. Lower FDR values in advancement group indicate a more posterior postoperative condylar position in comparison to the setback group.

